# The First-authored Papers Written by Chief Professors: Comparison before versus after Becoming a Professor

**DOI:** 10.31662/jmaj.2024-0095

**Published:** 2024-08-09

**Authors:** Shigeki Matsubara

**Affiliations:** 1 Department of Obstetrics and Gynecology, Jichi Medical University, Tochigi, Japan; 2 Department of Obstetrics and Gynecology, Koga Red Cross Hospital, Koga, Japan; 3 Medical Examination Center, Ibaraki Western Medical Center, Chikusei, Japan

**Keywords:** academic writing, first author, professor, workload, writing papers

## Abstract

I have long harbored the impression that professors of clinical medicine specialties tend to produce fewer first-authored papers after their professorship appointments, despite their prior output of first-authored papers. This humble experiment attempted in providing some suggestions for this issue. I identified 11 chief professors of the obstetrics and gynecology departments of Japanese medical universities who were appointed professorships during 201X − 201X + 3 (covering 4 years). The numbers of PubMed-indexed first-authored papers were retrieved: 7-4 years prior (Period 1), 3-0 years prior (Period 2), and 1-4 years after (Period 3) their professorship appointments. 1) The “total” number of papers in Periods 1, 2, and 3 was 38, 33, and 4, respectively. 2) The “median” number of papers written by an individual professor in Periods 1, 2, and 3 was 3, 2, and 0, respectively. 3) “Annual average” paper numbers per person before (Periods 1 + 2) versus after (Period 3) was 0.81 ((38 + 33)/(11 persons × 8 years)) and 0.09 (4/(11 × 4))/person/year, respectively. I did the same for “corresponding-authored papers or last-authored papers.” The results were as follows: 1) the “total” was 50, 74, and 143, respectively; 2) the “median” was 4, 5, and 7, respectively; and 3) the “annual average” was 1.41 versus 3.25/person/year. Thus, immediately after professorship appointments, the number of first-authored papers markedly decreased, although that of corresponding- or last-authored papers increased. The reason for this phenomenon may be multifactorial. However, societies should create an atmosphere where professors are relieved from excessive burdens and should be encouraged to engage in first-author paper writing as before if they desire. Societies want to hear professors’ own voices which enrich academic discourse. Although the present experiment targeted only Japanese obstetrics and gynecology professors for a limited time, I hope to provoke some discussion regarding paper writing and professorship.

## Professors Not Writing First-Authored Papers, True?

Publication is a professional responsibility for clinical doctors ^(1), (2)^. Having been immersed in medical academia for 4.5 decades, primarily in obstetrics and gynecology (OBGYN), I have long harbored the impression that university professors of clinical medicine specialties tend to produce fewer first-authored papers after their professorship appointments, despite their prior output. To my knowledge, there have been no publications addressing this issue. I investigated the validity of this impression. In this study, I did not aim to provide some conclusion, but I wished to provoke some discussion which has rarely been touched on.

## PubMed Examinations: The First-Authored Papers

I reviewed the homepages of the OBGYN departments at all Japanese medical universities. Excluding any branch schools and two recently established universities, I identified 11 professors who became chief professors over a consecutive 4-year period (201(X) − 201(X + 3)), with X = 1 representing 2011-2014. Directly describing X may inadvertently identify specific professors, posing confidentiality concerns given the topic’s sensitivity. Of the 11, seven were staff members of the corresponding department and were promoted to chief professors (internal promotions), while four were appointed from other institutions. Using PubMed, I determined the number of first-authored papers each professor published during three distinct periods, with each covering 4 years: 7-4 years prior (Period 1), 3-0 years prior (Period 2), and 1-4 years after (Period 3) their professorship appointments. I included all types of English papers but excluded Japanese language papers. I took care not to count papers written by different researchers with the same names.

The “total” number of papers in Periods 1, 2, and 3 was 38, 33, and 4, respectively. The “median” number of papers written by an individual professor in Periods 1, 2, and 3 was 3, 2, and 0, respectively. A comparison of the “annual average” paper numbers per person before (Periods 1 + 2) versus after (Period 3) was 0.81 ((38 + 33)/(11 persons × 8 years)) and 0.09 (4/(11 × 4))/person/year, respectively, which indicates a significant decline in the first-authored papers immediately after professorship appointments.

## PubMed Examinations: The Corresponding- or Last-Authored Papers

I performed the same search for the corresponding- or last-authored papers. For a paper where the first author is also the corresponding author, I counted it as a “first-authored paper” and did not count it as a “corresponding-authored paper,” avoiding duplicate counts. The “total” number was 50, 74, and 143, and the “median” number was 4, 5, and 7, in Periods 1, 2, and 3, respectively. The “annual average” number before (Periods 1 + 2) versus after (Period 3) was 1.41 ((50 + 74)/(11 persons × 8 years)) and 3.25 (143/(11 × 4)) person/year, respectively, indicating an increase in corresponding- or last-authored papers after professorship appointments. Corresponding- or last-authored papers in Period 3 did not differ between professors promoted within the corresponding department (*n* = 7) and those coming from outside (*n* = 4); 3.40 (95/(7 × 4)) versus 3.00 (48/(4 × 4)) person/year, respectively.

## Why Do First-Authored Papers Decline after the Appointment of a Professor?

The number of first-authored papers markedly decreased after professorship appointments, whereas the number of corresponding- or last-authored papers increased. I believe that three reasons contribute to the decline of first-authored papers after professorship appointments. First, professors face extensive workloads ^(2)^, including education, clinical duties, and administrative tasks, which limit the time for research, particularly paper writing; time for writing first-authored papers becomes further limited. Second, they may have less access to primary data as compared to their earlier career stages, as they allocate study themes to staff. This deprives them of the opportunity to write first-authored papers, particularly original articles. Third, there may be a prevailing expectation within society for professors in prioritizing the supervision and assistance to staff with paper writing, rather than engaging in first-authored publications by themselves ^(3)^. While some may say this is an excessive concern, I experienced such an atmosphere during my time as a professor. Indeed, the number of corresponding- or last-authored papers increased after professorship appointments.

## Medical Societies Should Do What?

My intention is not criticizing anyone for the decline of first-authored papers after a professorship appointment. I only wish to make some proposals. If professors themselves wish to continue writing first-authored papers and if society expects it, the following actions may help reverse this declining trend. They are to eliminate the factors of “why decline” described above. The first proposal is relieving the professors from excessive burdens, which requires some explanation. As previously described ^(2)^, university doctors, especially those in responsible positions, are expected to be “triple threat physicians” who excel in education, research, and clinical practice. Administrative tasks are added to the professorship, heavily burdening professors. Some possible solutions include job-sharing among department staff and the further introduction of nondoctor staff in assisting doctors and professors ^(2)^. A department as a whole, not an individual professor, should fulfill these “triple duties” ^(2)^. There is no need for professors to shoulder all these three burdens alone. Administrative duties are an important responsibility of professors, and thus, job-sharing for these tasks among staff is not straightforward. Personally, I believe that we should abandon the expectation that professors must always be there. For example, participation in all meetings (academic, social, and greeting events) may not always be necessary. Virtual participation should also be more widely accepted. Naturally, the implementation of these strategies depends on the specific situation; however, society should acknowledge them. We must consider that the concept of work-life balance applies not only to staff but also to professors. Second, medical societies should foster an atmosphere where professors are encouraged in engaging in first-authored paper writing as before if they desire. Third, our society, both academic and general, should emphasize that professors’ insights and expertise are communal assets, and the society wishes to hear them.

## Limitations but Some Merits at the Same Time

While I acknowledge there are some limitations to this humble experiment, it also holds some advantages. First, I only examined papers indexed in PubMed and did not assess the professors’ activities in medical meetings, both domestic and international. Second, the study focused solely on Japanese OBGYN professors. However, these limitations also offer some merits. The use of PubMed enabled an objective before-and-after comparison. While there may be variations across departments in paper writing practices, it is unlikely that OBGYN possesses unique features significantly affecting the productivity of professors’ first-authored papers. Thus, I believe that the present experiment may highlight at least some features of this issue, having some generalizability. My fundamental knowledge of Japanese OBGYN professors helped me in grasping accurate data (professors’ names and papers). Further research is necessary in determining if these findings are applicable to all professors worldwide.

## Good Effect for the Staff and Academia

The present study showed that after professorship appointments, even though the number of professors’ first-authored papers decreased, the number of corresponding- or last-authored papers increased, indicating the department’s overall paper-writing activity. Thus, someone may claim there is no problem with the decrease in first-authored papers. I think this view is an oversimplification. I believe that professors’ “current writing themselves” positively affects the education of staff. If professors do not have “direct primary data,” then writing Review, Opinion, or Viewpoint pieces is welcomed. The professors’ concepts or thoughts based on their long academic careers will surely touch the readers’ hearts and contribute to academic progress. This continuous writing will earn respect from staff members and foster a positive cycle within each university department, ultimately enhancing the progress of academic society.

Widening this view, I find some similarities between continuing writing and continuing clinical practice. I sometimes observed an instance. After a professorship appointment, a certain skilled surgeon-professor less frequently goes to the surgical theater. Some stop being the main operator but always become an assistant. I do not intend to say this is “wrong”: professors must educate staff by letting them become main operators. There may also be some other individual reasons for such behavior. However, department staff, especially younger generation doctors, usually respect their professors as “ongoing” (not “used to be”) writers and clinicians, even if they are not super writers or super clinicians. Professors’ fundamental stances and behaviors affect staff. Being a current writer and current clinician will give the younger staff a lifelong “yearning” to the professor and, ultimately, for academia. This atmosphere will nurture the department, university, and academic society. While this notion may seem old fashioned, the younger generation silently but continuously observes the “back” of the professor.

## Paper Writing for All

“Publication serves solely as a tool for promotion and its necessity diminishes thereafter”: I do not think so, and I believe that professors also do not think so. I believe in the enthusiasm of professors for academic writing. I believe writing papers is a pursuit which is relevant to all doctors, regardless of their professional rank or workplace ^(2), (3), (4), (5)^. Professors are no exception.

## Article Information

### Conflicts of Interest

None

### Acknowledgement

This is my personal view and does not represent the views of any institutes to which I am affiliated.

### Author Contributions

SM wrote the manuscript.

### Approval by Institutional Review Board (IRB)

Not applicable

### Data Availability

The data that support the findings of this study are available on request from the corresponding author. The data are not publicly available due to privacy or ethical restrictions.

### Patient Anonymity

Not applicable.

### Informed Consent

Not applicable.
